# Comparing options for females seeking permanent contraception in high resource countries: a systematic review

**DOI:** 10.1186/s12978-021-01201-z

**Published:** 2021-07-20

**Authors:** Rebecca Gormley, Brian Vickers, Brooke Cheng, Wendy V. Norman

**Affiliations:** 1 Faculty of Health Sciences, Simon Fraser University, Burnaby, BC Canada; 2 Faculty of Medicine, University of British Columbia, Vancouver, BC Canada; 3 Contraception & Abortion Research Team, Women’s Health Research Institute, BC Women’s Hospital and Health Centre, Vancouver, BC Canada; 4 Faculty of Public Health & Policy, London School of Hygiene & Tropical Medicine, London, UK; 5 Department of Family Practice, University of British Columbia, 320-5950 University Boulevard, Vancouver, BC V6T 1Z3 Canada

**Keywords:** Permanent contraception, Laparoscopic tubal ligation, Hysteroscopic tubal occlusion, Salpingectomy, Levonorgestrel intrauterine contraceptive, Systematic review

## Abstract

**Background:**

Multiple options for permanent or long-acting contraception are available, each with adverse effects and benefits. People seeking to end their fertility, and their healthcare providers, need a comprehensive comparison of methods to support their decision-making. Permanent contraceptive methods should be compared with long-acting methods that have similar effectiveness and lower anticipated adverse effects, such as the levonorgestrel-releasing intrauterine contraception (LNG-IUC). We aimed to understand the comparability of options for people seeking to end their fertility, using high-quality studies. We sought studies comparing laparoscopic tubal ligation, hysteroscopic tubal occlusion, bilateral salpingectomy, and insertion of the LNG-IUC, for effectiveness, adverse events, tolerability, patient recovery, non-contraceptive benefits, and healthcare system costs among females in high resource countries seeking to permanently avoid conception.

**Methods:**

We followed PRISMA guidelines, searched EMBASE, Pubmed (Medline), Web of Science, and screened retrieved articles to identify additional studies. We extracted data on population, interventions, outcomes, follow-up, health system costs, and study funding source. We used the Newcastle–Ottawa Scale to assess risk of bias and excluded studies with medium–high risk of bias (NOS < 7). Due to considerable heterogeneity, we performed a narrative synthesis.

**Results:**

Our search identified 6,612 articles. RG, BV, BC independently reviewed titles and abstracts for relevance. We reviewed the full text of 154 studies, yielding 34 studies which met inclusion criteria. We excluded 10 studies with medium–high risk of bias, retaining 24 in our synthesis. Most studies compared hysteroscopic tubal occlusion and/or laparoscopic tubal ligation. Most comparisons reported on effectiveness and adverse events; fewer reported tolerability, patient recovery, non-contraceptive benefits, and/or healthcare system costs. No comparisons reported accessibility, eligibility, or follow-up required. We found inconclusive evidence comparing the effectiveness of hysteroscopic tubal occlusion to laparoscopic tubal ligation. All studies reported adverse events. All forms of tubal interruption reported a protective effect against cancers. Tolerability appeared greater among tubal ligation patients compared to hysteroscopic tubal occlusion patients. No high-quality studies included the LNG-IUC.

**Conclusions:**

Studies are needed to directly compare surgical forms of permanent contraception, such as tubal ligation or removal, with alternative options, such as intrauterine contraception to support decision-making.

**Systematic review registration:**

PROSPERO [CRD42016038254].

**Supplementary Information:**

The online version contains supplementary material available at 10.1186/s12978-021-01201-z.

## Plain Language Summary

There are multiple options available to help people end their fertility: each option with accompanying benefits and risks. A comprehensive comparison of the benefits and risks of available options is important to support informed decision-making. We aimed to understand the comparability of laparoscopic tubal ligation, hysteroscopic tubal occlusion, bilateral salpingectomy, and a long-acting reversible contraceptive, the levonorgestrel-releasing intrauterine contraception (LNG-IUC), among females seeking permanent contraception in high resource countries.

We followed PRISMA guidelines for conducting systematic reviews. We assessed for risk of bias using the Newcastle–Ottawa scale, to ensure that we were including high-quality studies. We found high variability between the included articles, so we performed a narrative synthesis.

We identified 6612 articles and reviewed the full text of 154, of which 34 met our inclusion criteria. We further excluded 10 studies due to high risk of bias and included 24 articles in our synthesis. Most compared hysteroscopic tubal occlusion and/or laparoscopic tubal ligation. No included studies compared LNG-IUC to other methods. Most comparisons reported effectiveness and adverse events, with fewer reporting tolerability, patient recovery time, non-contraceptive benefits, and costs to the healthcare system. We found inconclusive evidence comparing the effectiveness of hysteroscopic tubal occlusion to laparoscopic tubal ligation. All options reported adverse events, and all forms of tubal interruption reported a protective effect against cancers.

There is insufficient research directly comparing surgical forms of permanent contraception, such as tubal ligation or removal, with alternative options, such as intrauterine contraception. High-quality studies are needed to support informed decision-making.

**Supplementary Information:**

The online version contains supplementary material available at 10.1186/s12978-021-01201-z.

## Introduction

Permanent contraception is the most common method of fertility control worldwide [1, 2]. Globally, nearly one in four females in high income countries use either intrauterine contraception or female sterilization [3]. Female permanent contraceptive methods are the fourth most commonly relied upon method for preventing pregnancy among people in Canada [4], and the second most common method in the United States [5]. Female permanent contraception is traditionally achieved using laparoscopic tubal ligation. However, in the last two decades, other methods to achieve permanent contraception have emerged including bilateral salpingectomy and the levonorgestrel-releasing intrauterine contraceptive (LNG-IUC), while Essure™, micro inserts used in hysteroscopic tubal occlusion, has been taken off the market in select countries [6–8]. In this review, we focus on comparing four methods used to achieve long-term or permanent contraception including: laparoscopic tubal ligation, hysteroscopic tubal occlusion, bilateral salpingectomy, and a long-acting contraceptive, the LNG-IUC.

Laparoscopic tubal ligation is traditionally achieved with the clipping, coagulation, or other blocking of the fallopian tubes to prevent sperm from travelling to an ovulated oocyte [2]. Hysteroscopic tubal occlusion is a procedure where micro inserts (i.e. Essure™) are placed in the fallopian tubes, and held by stainless steel inner and nickel-titanium outer coils. These coils encourage tissue growth, which after a few months blocks the fallopian tubes [9, 10]. It takes approximately three months for occlusion to occur, and during this time a woman is required to use alternate contraceptive methods. A post-procedure confirmation via ultrasound, hysterosalpingogram, or pelvic X-ray is required before a woman can discontinue alternative methods and the procedure is considered complete [11].

Bilateral salpingectomy is increasingly being considered as an alternative option to laparoscopic tubal ligation in several high resource countries such as Canada, the United States, Australia, and New Zealand [12–14]. With emerging evidence that ovarian cancer originates in the fallopian tubes [15], one Canadian province saw an increased trend in salpingectomy for female sterilization, from 0.4% of female sterilization procedures in 2008 to 33.0% in 2011 [16], with similar increases seen in Texas and New York over a similar time period [17]. At a Kaiser Permanente Northern California site, interval salpingectomies increased from 1.0 to 78.1% between 2011 and 2016 [18]. In June 2017, the Society of Obstetricians and Gynaecologists of Canada released a committee opinion that when counselling females seeking permanent contraception, physicians should discuss the protective benefit of tubal ligation against ovarian cancer, and the “fact that the removal of the fallopian tube may provide additional benefit” [14] with no additional side-effects over those with laparoscopic tubal ligation [15, 19]. In two Markov simulation models comparing bilateral salpingectomy and laparoscopic tubal ligation, bilateral salpingectomy was suggested to reduce ovarian cancer risk, contribute to additional quality years of life [20], and result in fewer ovarian cancer diagnoses [20], and fewer ovarian-cancer related deaths [21]; with a mean incremental cost of $152 per person [20]. Additional decades of follow up after salpingectomy are still needed to understand how closely reality will compare to this simulation.

The LNG-IUC is a long-acting reversible contraceptive that may be considered as an alternative to permanent contraception for females seeking to end their fertility. The insertion of the LNG-IUC has efficacy and effectiveness rates similar to tubal ligation [1–3, 5], as after insertion, it does not require maintenance. The LNG-IUC requires re-purchase and re-insertion every 5 or more years [22], may cause irregular bleeding [23], and in extremely rare cases, the device may migrate and cause uterine perforation [24]. However, this option may be attractive due to the avoidance of surgery and the faster recovery time compared to that required for other methods [25]. Therefore, with comparable effectiveness, fewer anticipated adverse events, and a similar ‘forgettable’ nature as available permanent contraceptive methods, the LNG-IUC should be considered an option for females seeking to end their fertility.

Despite the many options available, we were unable to find a guide for clinicians or for people seeking female permanent contraception that systematically compares available methods according to important outcomes, nor any that include comparable long-acting reversible contraception. Permanent contraception decision-making can be complex, and shared decision-making requires a comprehensive review of available options and relevant outcomes to make an informed choice that is aligned with each patients’ reproductive goals. Ultimately, the choice of which contraceptive method to use should be based on an informed understanding of not only effectiveness, but also any accompanying potential risks, additional benefits, tolerability, and recovery time expected for the patient.

### Objectives

We aimed to understand what is known from high quality studies about the comparability of permanent methods of contraception. We included laparoscopic tubal ligation, hysteroscopic tubal occlusion, bilateral salpingectomy, and insertion of the LNG-IUC among people seeking permanent female contraception in high resource countries.

Primary outcomes included:

- Effectiveness at preventing pregnancy
- Adverse events
- Tolerability
- Patient recovery; and
- Non-contraceptive benefits.

Secondary outcomes included:

- Length of procedure
- Costs to the healthcare system
- Eligibility
- Accessibility
- Follow-ups required to ensure completion or for safety monitoring.

Thorough definitions of study objectives are explained in the systematic review protocol [26].

## Methods

We followed PRISMA guidelines [27] for this analysis in accordance with the accompanying explanation and elaboration paper [28]. The PRISMA checklist is available in Additional file 1.

### Protocol registration

We pre-specified and previously published [26] objectives and analyses in a protocol registered on PROSPERO (CRD42016038254).

### Deviations from the protocol

In the previously published protocol, we did not explicitly state that we would remove studies which were assessed to be at high risk of bias. BC joined the project as an additional reviewer, who contributed to screening of the titles and abstracts. RG and BV were anticipated to do data extraction independently, and to compare results. As we will discuss, for this review RG extracted data for all included results, with BV extracting data from a sub-sample, which were compared for accuracy. Finally, in our protocol we noted that excluded studies would be listed in a table noting exclusion criteria. Instead, we have displayed the reasons for exclusion in the PRISMA flowchart for ease of presentation.

### Eligibility criteria

We included studies that met the following criteria:

- Population comprised of females of reproductive age (15–49), without major comorbidities;
- Prospective and retrospective cohort, case–control, or randomized control trial methodology;
- The paper was published in a peer reviewed journal in English;
- The analysis took place in high resource countries as defined by the World Bank Country and Lending groups [29];
- The interventions included comparisons of any two or more of: laparoscopic tubal ligation, hysteroscopic tubal occlusion, bilateral salpingectomy, or insertion of the LNG-IUC, and/or controls;
- The outcomes assessed included at least one of the following: effectiveness, adverse events, tolerability, non-contraceptive benefits, patient recovery, accessibility, length of the procedure, follow-ups required, eligibility, or costs to the healthcare system.

We excluded studies with these characteristics:

- A case study or case series design;
- Conducted outside of high resource countries;
- Interventions included concomitant procedures.

### Information sources

We searched EMBASE, Pubmed (Medline), and Web of Science using a combination of MeSH terms and key words related to hysteroscopic tubal occlusion, laparoscopic tubal ligation, the LNG-IUC, and bilateral salpingectomy. We also reviewed the references of relevant articles. We did not set date restrictions.

### Search strategy

We downloaded selected articles in Mendeley Desktop 1.19.3 software (Elsevier, 2008) for further assessment and handling, including study selection. We consulted librarians to create our search strategy, which is available online: http://med-fom-cart-grac.sites.olt.ubc.ca/files/2016/05/Search-Strategies-Librarian-edit.docx.

The search strategy was built between April and May 2016. An initial search was performed on May 16th, 2016. We performed an updated search of the literature, using the same search strategy as outlined above, on January 30th, 2019.

### Study selection

Three authors (RG, BC, BV) independently reviewed titles and abstracts of initial articles based on relevance. RG reviewed all identified articles, and BC and BV each reviewed a subset of articles. After comparison of articles for relevance based on titles and abstracts, RG reviewed full text articles for inclusion or exclusion, noting reasons for exclusion.

### Data collection process

RG created the data extraction form and initially pilot-tested the form on a randomly selected subset of studies to determine comprehensiveness. We extracted data from each study that met the inclusion criteria including: population, intervention, comparisons, outcomes, and study design (PICOS); follow-up period; and funding source for the study, where available.

RG extracted data from all relevant articles, and BV independently extracted data from a sample of articles. We compared the data extraction forms for accuracy. Any discrepancies were adjudicated by the senior author (WVN).

### Data items

We defined all data items, including definitions of the variables sought, in detail in our protocol [26].

### Risk of bias in individual studies and across studies

We used the Newcastle Ottawa Scale (NOS) to determine risk of bias for cohort and case–control studies [30]. We assessed risk of bias for each included study, and presented the results in a table stratified by study design. As the GRADE guidelines suggest, in evaluating a large body of evidence, it is important to consider risk of bias across outcomes. Where an outcome is reporting data from studies that are at high risk of bias and at low risk of bias, authors may consider synthesizing only those at low risk of bias [31]. Therefore, we excluded any articles assessed to be at medium–high risk of bias (NOS score < 7).

We assessed the cumulative risk of bias based on the risk of bias found in individual studies, along with careful consideration of any outcome reporting bias, incomplete study data, or overall quality of the evidence presented and synthesized.

### Synthesis of results

We aimed to perform a network meta-analysis, but heterogeneity was assessed as substantial (I^2^ ≥ 80%), with wide variability in outcome reporting that precluded a valid pooling of results. Therefore, we undertook a narrative synthesis in accordance with the guidelines of the Cochrane Consumers and Communication Review Group [32], by using text to summarize the overall effects and variations in studies, and synthesis across included studies to identify the patterns and interpretations of overall findings [33]. First, we summarized the main outcomes and effects for each study and grouped by intervention comparison and outcome. Next, we assessed differences to explain the patterns of effect, by considering variability in the study designs, in populations and settings, and the outcome measures used [32]. We organized the final narrative synthesis by outcome, describing the similarities, differences, and patterns of results.

## Results

### Study selection

**Fig. 1:**
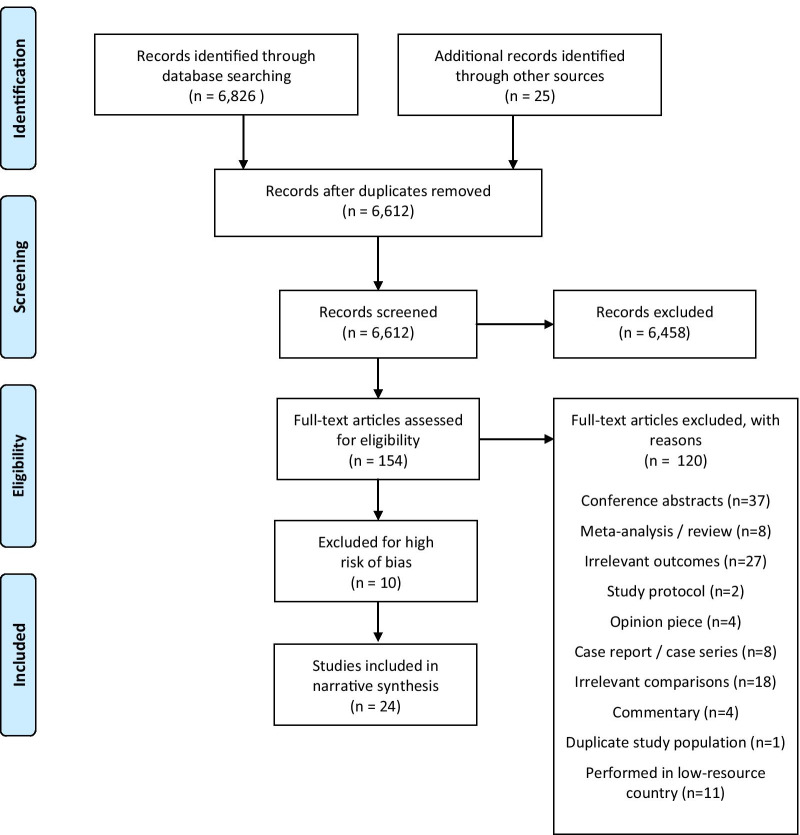
PRISMA flow diagram

Figure 1 details our study selection process, including reasons for exclusion, following PRISMA guidelines [27]. Our database search of EMBASE, Pubmed (Medline), and Web of Science revealed 6,826 documents, and we identified an additional 25 documents screening references of relevant articles. After we excluded duplicates (239), RG, BC, BV reviewed titles and abstracts based on relevance. RG reviewed all 6,612, and BC and BV independently reviewed 1,647 and 5,088 studies respectively. Of these, we excluded 6,458 because they did not meet the inclusion criteria. RG reviewed the full text of the remaining 154 studies. BV reviewed 30 full text studies and compared them with the relevant articles assessed by RG to check for accuracy. We extracted data and assessed risk of bias for 34 studies. We found high risk of bias in 10 studies, leaving a total of 24 studies included in the narrative synthesis.

### Study characteristics

Included studies compared laparoscopic tubal ligation vs. control (n = 2), hysteroscopic tubal occlusion vs. laparoscopic tubal ligation (n = 12), laparoscopic tubal ligation vs. bilateral salpingectomy (n = 6), laparoscopic tubal ligation vs. bilateral salpingectomy vs. control (n = 3), hysteroscopic tubal occlusion vs. bilateral salpingectomy vs. laparoscopic tubal ligation (n = 1). Our search revealed only one study which included outcomes with the LNG-IUC, however it was excluded due to high risk of bias.

People who had severe comorbidities were excluded. All studies were pairwise comparisons of females of reproductive age, who underwent an interval contraceptive procedure (at a time not related to a pregnancy) including either laparoscopic tubal ligation, hysteroscopic tubal occlusion, bilateral salpingectomy, or were selected as a control comparison.

The characteristics and results of all included studies can be found in Table 1. Six studies compared effectiveness [34–39]; 15 assessed adverse effects [17, 18, 34, 35, 38–48]; three compared patient recovery [18, 35, 44]; five compared non-contraceptive benefits, primarily the reduction of cancer risk [43, 49–52]; six compared tolerability [34, 35, 37–39, 52]; four compared costs to the healthcare system; [38, 53–55] and seven compared length of procedures [17, 18, 38, 44, 47, 48, 55]. No included studies compared accessibility, eligibility, or follow-up required. The majority of studies (n = 20) were observational cohorts, and the rest were case–control studies (n = 4). The studies were conducted in the United States (n = 13), Canada (n = 2), the UK (n = 2), France (n = 2), Denmark (n = 1), Spain (n = 1), Sweden (n = 1), Finland (n = 1), and Australia (n = 1). All included studies were published between 2003 and 2019. Enrolment of females of reproductive age (15–49) occurred between 1966 and 2016, with significant variations in follow-up ranging from 2 weeks to 44 years.

**Table 1:** Characteristics of studies, including risk of bias, included in comparing female permanent contraception options in high resource countries: a systematic review (n = 34)

First author, year	Study period	N	Country	Population (mean/median age)	Intervention	Comparison	Outcomes reported	Study design	Follow up period	Funding	Risk of bias
Abbuhl, 1997	1990–1991	24 = LTL 182 = Control	US	30.8 vs. 24.1	LTL	No sterilization	Adverse events	Retrospective cohort study	Not reported	Not reported	Medium
Antoun, 2017	2005–2015	1085 = HTO 2412 = LTL	UK	36.1 vs. 35.6	HTO	LTL	Effectiveness Adverse events Tolerability	Observational Cohort	1–10 years	Not reported	Low
Bouillon, 2018	2010–2015	71,303 = HTO 34,054 = LTL	France	41.5 vs. 40.8	HTO	LTL	Effectiveness Adverse events Tolerability Patient recovery	Cohort, nation-wide database	1–3 years	Not reported	Low
Carmona, 2003	1994	31 = LTL 31 = Control	Spain	36.4 vs. 36.1	LTL	No sterilization	Adverse events	Case–control	5 years	Not reported	Low
Carney, 2017	2010–2012	12,031 = HTO 7286 = LTL	US	37.0 vs. 35.8	HTO	LTL	Costs to Healthcare System	Retrospective Cohort	6 months	Supported by Bayer HealthCare	Low
Conover, 2015	2005–2012	26,927 = HTO 44,948 = LTL	US	37.8 vs. 36.6	HTO	LTL	Adverse events	Prospective Cohort (administrative claims)	275 days HTO 283 days LTL	Investigator funding from Agency for Healthcare Research and Quality, and HIH, National 7Heart Lung & Blood Institute	Low
Duffy, 2005	Not reported	59 = HTO 24 = LTL	UK	35.1 vs. 36.1	HTO	LTL	Effectiveness Adverse Tolerability Length of Procedure	Cohort controlled comparative trial	3 months	Not reported	Medium
Falconer, 2015	1973–2009	34,433 = BS 81,658 = LTL 5,449,119 = Unexposed	Sweden	35.7 vs. 37.9 vs. 35.9	BS LTL	No sterilization	Non-contraceptive benefits	Population based cohort study	18 years BS 21.4 years LTL 23.1 years no sterilization	Stockholm City Council	Low
Fernandez, 2014	2006–2010	39,169 = HTO 70,108 = LTL	France	41 vs. 40	HTO	LTL	Effectiveness	Retrospective cohort (hospital discharge)	1–4 years	Conceptus (manufacturer of Essure) provided CB, LL expenses for this study	Low
Franchini, 2009	2005–2007	24 = LTL 25 = HTO	Italy	Not reported	HTO	LTL	Patient Recovery Cost to the healthcare system Length of procedure	Case–control Activity based cost management	Not reported	Not reported	Medium
Gaitskell, 2016	1996–2001	294,724 = LTL 984,059 = Control	UK	55.4 vs. 56.3	LTL	No sterilization	Adverse events Non-contraceptive benefits	Prospective cohort study	13.8 years LTL 13.8 years no sterilizations	Cancer Research UK, UK Medical Research Council	Low
Greisman, 1991	1981–1987	22 = Ectopic with LTL 268 = Ectopic no LTL	Canada	33.5	LTL	No sterilization	Adverse events	Case–control	Not reported	Not reported	Medium
Hanley, 2018	2008–2014	19,424 = LTL 5839 = BS	Canada (BC)	35.3 vs. 36.4	LTL	BS	Adverse events	Retrospective cohort study	2 weeks	Canadian Cancer Society Research Institutes, CIHR, UBC Hospital Foundation	Low
Hopkins, 2007	2003–2004	43 = HTO 44 = LTL	US	37.2 vs. 37.7	HTO (operating room)	LTL	Costs to the healthcare system Length of procedure	Retrospective cohort study	Not reported	Not reported	Low
Jokinen, 2017	2009–2014	5631 = HTO 4425 = LTL	Finland	38.0 vs. 35.5, 37.8	HTO	LTL	Effectiveness Tolerability	National Register, study linkage	Not reported	Not reported	Low
Kjer, 1990	1978–1981	10,104 = LTL 847,012 = Control	Denmark	NA	LTL	No sterilization	Effectiveness Adverse events	Case–control	4–7 years	Not reported	Medium
Kim, 2019	2013–2016	180 = BS 274 = LTL	US	32.3 vs. 33.1	LTL	BS	Adverse events Length of procedure	Retrospective cohort study	Not reported	Not reported	Low
Lessard-Anderson, 2014	1966–2009	194 = Cases 388 = Controls	US	61.4 vs. 61.4	BS LTL	Matched control	Non-contraceptive benefits	Case–control (nested)	44 years	Not reported	Low
Levie, 2005	Unspecified	Unspecified	US	Unspecified	HTO (office setting)	LTL (surgical)	Costs to the healthcare system	Case–control Cost comparison analysis	Not reported	Not reported	Low
Madsen, 2015	1982–2011	13,241 = Cases (ovarian cancer) 194,689 = Controls (ovarian cancer) 3605 = Cases (ovarian tumour) 53,322 = Controls (ovarian tumour)	Denmark	Each case (30–84, no previous cancer) matched with 15 randomly selected matched on date of birth from Civil Registration	BS, LTL	No sterilization	Non-contraceptive benefits	Case–control (register-based)	Not reported	Danish Cancer Society Scientific Board	Low
Malacova, 2014	1990–2010	278 = HTO 20,429 = LTL 553 = BS 22,295 = unspecified	Australia	18–44	HTO, BS, LTL	Unspecified destruction of tubes	Adverse events	Retrospective cohort study	Up to 15 years	Not reported	Low
Mao, 2019	2005–2016	10,143 = HTO 53,206 = LTL	US (New York)	34.9 vs. 34.1	HTO	LTL	Tolerability Non-contraceptive benefits	Observational cohort	7 years	Not reported	Low
Mao, 2015	2005–2013	8048 = HTO 44,278 = LTL	US (New York)	54.9% vs. 55.3% between 30–39	HTO	LTL	Effectiveness Adverse events Tolerability Length of procedures Costs to healthcare system	Observational, Population based cohort study	1 year	UO1 grant (NIH- 1U01FD004494-01). MDEpiNet Science and Infrastructure Centre. JM is an analyst within the Weill Cornell Medical College (WCMC) Patient Centered Comparative Effectiveness Program and the Medical Device Epidemiology Network’s (MDEpiNet) Science and Infrastructure Center: AS is the director of the Center)	Low
McAlpine, 2014	2008–2011	1569 = BS 13,719 = LTL	Canada (British Columbia)	36.0 vs. 34.8	BS	LTL	Patient recovery Adverse events Length of procedure	Retrospective cohort study	Not reported	Vancouver General Hospital and University of British Columbia Hospital Foundation and the British Columbia Cancer Foundation	Low
Niblock, 2014	2008–2011	60 = HTO 25 = LTL	UK	36.5 vs. 35.1	HTO	LTL	Effectiveness Tolerability Adverse events Patient Recovery	Retrospective chart review	6–50 months	Not reported	Medium
Perkins, 2016	2007–2013	27,724 = HTO 42,391 = LTL	US	37.4 vs. 36.7	HTO	LTL	Effectiveness Adverse events Tolerability	Retrospective cohort study	2.25 years HTO 2.33 years LTL	Not reported	Low
Powell, 2017	2011–2016	1483 = BS 2229 = LTL	US (Northern California)	36 vs. 36	BS	LTL	Adverse events Patient recovery Length of procedure	Retrospective cohort study	5 years	Not reported	Low
Rulin, 1993	Not reported	500 = LTL 466 = Comparison	US (3 hospitals: Pittsburgh, Atlanta, NY)	28 vs. 27	LTL	No sterilization	Adverse events	Cohort	3–4.5 years	2 R01 HD 19398-04 National Institutes of Health	Medium
Steward, 2017	2009–2012	3929 = HTO 10,875 = LTL	US	31.8 vs. 30.4	HTO	LTL	Adverse events	Retrospective cohort study	24 months	Financial support from Bayer for the study, and employees involved in design, execution, analysis, reporting of this paper	Low
Syed, 2007	2003–2004	20 = LTL 20 = HTO	US – Staten Island Uni	42.5 vs. 38	HTO	LTL	Adverse events Patient Recovery Length of procedure	Cohort study	6 months	Not reported	High
Theil, 2008	HTO = 2005–2006 LTL = 2001–2004	108 = HTO 104 = LTL	Regina, Canada	36.8 vs. 33.4	HTO	LTL	Tolerability Length of procedure Costs to the healthcare system	Retrospective cohort study	Not reported	Not reported	Medium
Trussel, 1995	1991–1993	20,000 public payments from commercial insurers	United States	Not reported	LTL	LNG-IUC	Costs to the healthcare system	Cohort	Not reported	Wyeth-Ayerst Laboratories	Medium
Westberg, 2017	2011–2015	81 = BS 68 = LTL	US (UC Davis Medical Center)	35.6 vs. 36.2	BS	LTL	Adverse events Length of procedure	Retrospective cohort study (chart review)	30 days	Not reported	Low
Zerden, 2018	2014–2015	13 = BS 5 = Current LTL 22 = Historical LTL	US	35.0 vs. 34.6 vs. 34.9	BS	LTL (current and historical)	Adverse events Length of procedure	Cohort study	Not reported	Ligasure Instruments (bipolar sealing device) donated by Medtronic/Covidien	Low

### Risk of bias within studies

We excluded studies where risk of bias was determined to be medium to high (NOS 0–6) in at least one domain of assessment of risk, largely due to the observational study designs, non-random allocation of interventions, and differences in baseline characteristics between comparator groups. Our assessment of risk of bias for each study can be found in Additional file 2.

### Results of individual studies

Results of individual studies can be found in Table 1.

### Narrative synthesis of results

#### Effectiveness

Six studies reported the rate of pregnancy, all of which were cohort studies comparing hysteroscopic tubal occlusion and laparoscopic tubal ligation [34–39]. Among the included studies, there was a wide range of follow-up time to assess effectiveness (from 1 year to a maximum of 10 years) [34] and significant variance in directionality and strength of the outcome; this likely explains the considerable heterogeneity observed.

Three analyses found no significant difference in the risk or reported number of unintended pregnancies between laparoscopic tubal ligation and hysteroscopic tubal occlusion [34, 37, 38]. A retrospective cohort in the United States found that the cumulative rate of pregnancy was 1.02 pregnancies per 100 person years after hysteroscopic tubal occlusion and 0.88 pregnancies per 100 person years after laparoscopic tubal ligation (p = 0.003). Patients who underwent hysteroscopic tubal occlusion were at 1.2 times higher risk of becoming pregnant compared to those who underwent laparoscopic tubal ligation [aHR 1.20 (95% confidence interval 1.09–1.33)]. [39].

Two studies, both conducted in France, found a higher risk of pregnancy among laparoscopic tubal ligation than hysteroscopic tubal occlusion [35, 36]; however in one study, this difference was only significant at 1 year [aHR 0.70 (0.53–0.92)], but not at 3 years [aHR 1.04 (0.83–1.30)] [35].

#### Adverse effects

Fifteen studies assessed adverse effects. Results are organized by comparison: six compared hysteroscopic tubal occlusion and laparoscopic tubal ligation [34, 35, 38–41], two compared laparoscopic tubal ligation and a control [42, 43], five compared laparoscopic tubal ligation and bilateral salpingectomy [17, 18, 44, 45, 48], one compared hysteroscopic tubal occlusion, laparoscopic tubal ligation, and bilateral salpingectomy with controls [46], and one compared bilateral salpingectomy with laparoscopic tubal ligation and with historical matched controls [47].

##### Hysteroscopic tubal occlusion vs. laparoscopic tubal ligation

Three studies [34, 40, 41] found no statistically significant difference in rates of adverse effects including abnormal uterine bleeding, pelvic pain, or opioid managed pain between the two interventions, including at 6 or 12 months post-procedure. A significantly lower risk of chronic pelvic pain and risk of hysterectomy was found among women undergoing hysteroscopic tubal occlusion at 24 months post-procedure [41].

Hysteroscopic tubal occlusion was associated with a lower risk of surgical complications than laparoscopic tubal ligation [aOR 0.18 (0.14–0.23)] [35] and a lower risk of iatrogenic complications after surgery [OR 0.35 (0.20–0.61)] [38]. Higher rates of gynecological complications were found in hysteroscopic tubal occlusion patients compared to laparoscopic tubal ligation patients [35, 39], including menstrual dysfunction [aHR 1.23 (1.20–1.27)] [39]. However, pelvic pain incidence was found to be significantly lower in hysteroscopic tubal occlusion patients compared to laparoscopic tubal ligation patients [21.0% compared with 25.6% at 2 years, aHR 0.83 (0.80–0.85)] [39].

##### Laparoscopic tubal ligation vs. comparison

Compared to controls, included studies did not find a statistically significant change in menstrual cycle after undergoing laparoscopic tubal ligation [42].

One study found an increase in risk for anal cancer among those who underwent laparoscopic tubal ligation compared to those who did not [RR 1.34 (1.11–1.63)]; however, no associations between laparoscopic tubal ligation and risk of endometrium, breast, cervix, or colorectal cancers, nor all cancers combined, were significant [43].

##### Laparoscopic tubal ligation vs. bilateral salpingectomy

Three studies found that there was no significant difference when comparing risk of readmission, blood transfusion, or intraoperative complications between laparoscopic tubal ligation and bilateral salpingectomy [17, 18, 44], nor any difference in post-procedure physician visits for surgical infection or complication [45]. No significant differences in complications were found when assessing both immediate (2.9% vs. 2.5%, p = 1.0) and short-term (within 30 days) adverse events (14.7% vs. 4.9%, p = 0.51) among people undergoing laparoscopic tubal ligation and bilateral salpingectomy, respectively [48]. However, there was a higher risk among people who underwent bilateral salpingectomy who required prescription analgesic use after surgery compared to those who underwent laparoscopic tubal ligation [(aOR 1.21 (1.14–1.29)] [45].

##### Hysteroscopic tubal occlusion vs. laparoscopic tubal ligation vs. bilateral salpingectomy vs. controls

One study performed a retrospective cohort study using administrative data to assess risk of ectopic pregnancy among people who underwent surgical sterilizations including bilateral salpingectomy, laparoscopy with Filshie clip, minilaparotomy, laparotomy, and hysteroscopic tubal occlusion using Essure™ compared to an unspecified destruction or occlusion of fallopian tubes [46]. Hazard ratios for ectopic pregnancy did not remain significant for laparoscopy with Filshie clip, minilaparotomy, and laparotomy, and there were no ectopic pregnancies reported for bilateral salpingectomy nor hysteroscopic tubal occlusion with Essure™ [46].

##### Bilateral salpingectomy vs. tubal ligation vs. historical controls

When comparing bilateral salpingectomy and laparoscopic tubal ligation to historical controls, there was no significant difference when comparing estimated median blood loss between bilateral salpingectomy (5 ml) with laparoscopic tubal ligation current (7 ml, p = 0.18) or historical (10 ml, p = 0.31) controls [47].

#### Patient recovery

Two studies comparing laparoscopic tubal ligation and bilateral salpingectomy found no significant difference in terms of length of hospital stay, although there was a wide variance in reported length between the two studies (median of 1.8 h [18] to 1.31 days [44]).

One study found that women who underwent hysteroscopic tubal occlusion required fewer sick days compared to women who underwent laparoscopic tubal ligation at 1 year (5.90 days vs. 6.50 days, p < 0.001) and at 3 years (28.3 vs. 32.3, p < 0.001). [35].

#### Non-contraceptive benefits

Five studies measured non-contraceptive benefits, primarily assessing preventative benefits in reducing the risk of developing various cancers. Three compared bilateral salpingectomy or laparoscopic tubal ligation against controls [49–51], one compared laparoscopic tubal ligation against controls [43], and the last compared hysteroscopic tubal occlusion and laparoscopic tubal ligation directly [52]. Two were case–control studies [50, 51] while the rest were cohorts using large administrative databases [43, 49, 52].

Overall reduction in ovarian cancer risk [RR 0.80 (0.76–0.85)], peritoneal cancers [RR 0.81 (0.66–0.98)], and cancers of the fallopian tube [RR 0.60 (0.37–0.96)] was observed in laparoscopic tubal ligation patients when compared to matched controls [43].

Both laparoscopic tubal ligation and bilateral salpingectomy had protective effects against cancers when compared to a matched control. Both laparoscopic tubal ligation [aHR 0.72 (0.64, 0.81)] and salpingectomy [aHR 0.65 (0.52–0.81)] had protective effects against ovarian or tubal cancer compared to females who did not have any surgical intervention [49]. A sub-analysis found that bilateral salpingectomy had a greater reduction in risk than unilateral salpingectomy [aHR 0.35 (0.17–0.73) vs. aHR 0.71 (0.56–0.91) respectively], although data distinguishing laterality was only available up to 1996 [49]. Similarly, people who underwent laparoscopic tubal ligation or bilateral salpingectomy had reduced odds of developing epithelial ovarian cancer ([OR 0.87 (0.78–0.98)] and [OR 0.58 (0.36–0.95)], respectively) compared to matched controls [50].

An age-matched case–control study found that when adjusted, a history of any tubal sterilization proved to have a statistically non-significant odds ratio of reducing the risk of developing epithelial ovarian cancer [OR 0.59 (0.29–1.17)] compared to matched controls [51]. Further analyses comparing the effect of bilateral salpingectomy against matched controls, non-excisional techniques, and partial salpingectomy also remained statistically insignificant [OR 0.22 (0.03–1.87)] [51]. Similarly, Mao et al. found no difference in the incidences of gynecologic cancer [(0.1% vs. 0.1%, HR 2.63 (0.70–9.91)] or other cancers [(1.2% vs. 1.3%, HR 1.03 (0.78–1.36)] after initial hysteroscopic tubal occlusion compared with laparoscopic tubal ligation [52].

#### Tolerability

Nine studies assessed the ability to perform the intended method without requiring other procedures to fix the procedure due to an unsuccessful first attempt or to perform a second procedure to achieve permanent contraception. All studies compared the tolerability of hysteroscopic tubal occlusion with laparoscopic tubal ligation with a wide variation in follow-up time, from an average of 30 days [34] to 7 years. [52].

Six studies found a significantly higher risk of re-operation among those who underwent hysteroscopic tubal occlusion compared to laparoscopic tubal ligation [34, 35, 37–39, 52], with studies reporting an increased adjusted hazard ratio from 2.05 [39], to 3.26 (1 year post-procedure) [35] and tenfold the odds (1 year post-procedure) [38]. The increased risk of re-operation remained after a 3-year follow up [aHR 1.62 (1.51–1.73)] [35].

#### Accessibility

We did not find an eligible study that systematically measured or compared the out-of-pocket costs for the procedure, wait times, or the locations where the procedure can be performed.

### Secondary objectives

#### Eligibility

We did not find an eligible study that systematically measured or compared eligibility for the procedures.

#### Follow-up required

We did not find an eligible study that compared the number of follow-up visits needed, or required, to ensure that the method was completed or for safety monitoring.

#### Costs to the healthcare system

Four studies measured costs to the healthcare system by index cost. All studies compared total costs between hysteroscopic tubal occlusion and laparoscopic tubal ligation, either reporting the mean or the median index costs per patient when undergoing each procedure. Three studies found that hysteroscopic tubal occlusion was less costly to perform than laparoscopic tubal ligation [53–55], with total costs for hysteroscopic tubal occlusion ranging between $1646 [54] and $3964 [53]. Median total costs for laparoscopic tubal ligation ranging between $2880 [55] and $5163 [53]. One study found that total medical and prescription costs ($7093 vs. $7568, p < 0.0001) and procedure-related costs ($4971 vs. $5407, p < 0.0001) were lower among women who underwent hysteroscopic tubal occlusion compared to tubal ligation [53]. However, costs related to complications or failures were higher with hysteroscopic tubal occlusion compared to laparoscopic tubal ligation ($272 vs. $176) [54]. One study found higher total charges for hysteroscopic tubal occlusion compared to laparoscopic tubal ligation (median $7832 vs. $5068, p < 0.01) [38].

#### Length of the procedure

Seven studies compared the length of the procedure, with four comparing bilateral salpingectomy and laparoscopic tubal ligation, two comparing laparoscopic tubal ligation and hysteroscopic tubal occlusion, and one comparing bilateral salpingectomy with tubal ligation and historical controls.

Four studies found that the bilateral salpingectomy procedure took significantly longer than laparoscopic tubal ligation to complete (3–11 min longer) [17, 18, 44, 48] Median/mean surgical times ranged between 33 [18] and 44 [48] min for bilateral salpingectomy, and between 30 [18] and 38 [48] min for laparoscopic tubal ligation. Overall median operative times were similar between bilateral salpingectomy and tubal ligation (study and historical controls) [47].

Laparoscopic tubal ligation procedure took significantly longer (means and medians ranging between 27 and 52 min) than hysteroscopic tubal occlusion (means and medians ranging between 18–36 min) [38, 55].

### Risk of bias across studies

Overall, the cumulative evidence presented remains at low to medium risk of bias. Due to the observational study designs used, we found that there were significant sociodemographic differences between comparator groups that were not able to be adjusted for. Some studies did not fully report their patient demographic, leading to questions about comparability. With high heterogeneity found, our interpretation of evidence must be balanced and cautious. Our conclusions focus on comparisons between laparoscopic tubal ligation and hysteroscopic tubal occlusion, and we described tentative conclusions with other comparisons.

### Additional analysis

We did not conduct any additional analyses.

## Discussion

### Summary of evidence

There is insufficient data to compare available options for people seeking female permanent contraception, especially comparing to the LNG-IUC. Most studies eligible for our review compared laparoscopic tubal ligation to hysteroscopic tubal occlusion using Essure™ micro inserts, which is no longer available for use in some jurisdictions [6–8]. Most comparisons reported on effectiveness and adverse events; fewer reported tolerability, patient recovery, non-contraceptive benefits, and/or healthcare system costs. No comparisons reported accessibility, eligibility, or follow-up required.

The majority of studies in our review comparing hysteroscopic tubal occlusion and laparoscopic tubal ligation did not find a significant difference in effectiveness. However, for hysteroscopic tubal occlusion, success of effectiveness relied on participants using another form of contraception, or abstinence, before tubal occlusion could be confirmed with a hysterosalpingogram and correct bilateral placements of the micro inserts. Other options, including laparoscopic tubal ligation, bilateral salpingectomy, and insertion of the LNG-IUC are immediately effective. Although studies that assessed the effectiveness of the LNG-IUC were not included, other reviews have demonstrated the high efficacy of this method, with a cumulative pregnancy rate of 0.5 per 100 users [23], which appears comparable to laparoscopic tubal ligation [56].

Non-contraceptive benefits primarily looked at protective effects against various types of cancers. While the magnitude of the protective effect differed between methods of permanent contraception, it appears that undergoing some form of tubal interruption—whether it be occlusion, ligation, or removal—has a protective effect against several types of gynecologic cancers. In separate reviews, the LNG-IUC is also suggested to have protective effects against gynecological cancers [57], as well as menorrhagia, endometriosis, adenomyosis, and fibroids [23]. Longer term cohort studies will be required to effectively compare these protective effects among all available options for female permanent or long acting contraception.

All options for female permanent have risks of adverse effects; however, our review did not find significant differences in opioid managed pain, pelvic pain, menstrual dysfunction, or intraoperative complications when comparing surgical methods and/or controls. One study found that hysteroscopic tubal occlusion patients had a lower risk of post-procedure hysterectomy 24-months post-procedure [41], but the strength of the evidence is diminished with potential bias in funding from Bayer, the company that created the Essure™ device. Multiple studies have found an association between an increased risk of anal cancer and a history of laparoscopic tubal ligation [43, 58]. Although we did not find articles assessing adverse events of the LNG-IUC to include in our review, previous studies found minimal adverse effects, with some attributed to the device itself such as dysmenorrhea or irregular bleeding, or to the levonorgestrel such as weight gain [23]; although there is conflicting evidence with weight gain due to levonorgestrel [59]. More serious complications, such as uterine perforation, are found to occur rarely (estimated 2.6 per 1000 insertions) [24]. A systematic review comparing complication rates between laparoscopic tubal ligation and bilateral salpingectomy for sterilization found no significant differences in blood loss, perioperative complications, or rehospitalizations [60]. Upcoming research comparing salpingectomy and tubal ligation found no difference in time to first physician visit related to menopause between patients [61].

Laparoscopic tubal ligation was found to be more tolerable than hysteroscopic tubal occlusion using Essure™ micro inserts, with patients undergoing hysteroscopic tubal occlusion requiring higher rates of re-operation to complete or to fix the previous contraceptive method [34, 35, 37–39, 52]. Although not included within our review, separate research suggests high tolerability among LNG-IUC users, including nulliparous females [62]. Spontaneous expulsion of the LNG-IUC is uncommon, with an overall crude incidence of 9.6% [63]. Spontaneous expulsion is higher in patients with adenomyosis, uterine leiomyoma, heavy menstrual bleeding, and dysmenorrhea [63]. Premature removal of the LNG-IUC has been found to be associated with excessive bleeding, pelvic infections, pain, depression, and recurrent infections; however even in the reported study, continuation rates, especially among older cohorts, were high [64].

Patient recovery was assessed by length of hospital stay between salpingectomy and tubal ligation, where no significant difference was found. Patients undergoing laparoscopic tubal ligation reported more sick days at 1 and 3 year follow ups compared to hysteroscopic tubal ligation [35]. Considerations including patient satisfaction, time to return to work, and other factors need to be explored further. Separate reviews have found have high patient satisfaction after the insertion of the LNG-IUC, and an almost immediate recovery time [65–67].

Lastly, we considered costs to the healthcare system. While three out of four studies assessing hysteroscopic tubal occlusion and laparoscopic tubal ligation found that hysteroscopic tubal occlusion was significantly less costly to perform than laparoscopic tubal ligation [53–55], costs related to complications or failures were higher after hysteroscopic tubal occlusion [54]. Costs to the healthcare system also should balance preventative costs, such as savings per life-year gained with prevention of cancer cases. No studies calculated preventative cost-savings accompanying non-contraceptive benefits in each method, despite evidence that laparoscopic tubal ligation [43, 50, 51], salpingectomy [49, 50], and the LNG-IUC [68] provide potential cancer risk reduction. Three Markov models predicted significant cost-effectiveness when bilateral salpingectomy is employed in place of laparoscopic tubal ligation in terms of ovarian cancer prevention and life-years gained [20, 21, 69]. Future patient cohort studies will be needed to determine savings realized in practice.

### Implications

People seeking to end their fertility need to be able to make an informed decision on the range of available options with their healthcare providers. However, there is insufficient evidence to compare surgical options, such as tubal ligation or bilateral salpingectomy, with other non-surgical options such as intrauterine contraception that offer similar effectiveness. Beyond ending fertility, other factors that will influence and inform a person’s choice for what method is best for them. To make a rigorous comparison, there is a need for high-quality research to be performed with a broader range of options.

### Limitations

We found significant heterogeneity between the included articles. This high heterogeneity is likely driven by their methodological diversity and observational study designs, which did not allow for randomized allocation of participants. Therefore, significant differences in study population likely existed between the different intervention types, such as age, socioeconomic status, or underlying health conditions that were not excluded as a major comorbidity. Follow-up times varied widely between the included studies, with some only allotting minimal weeks for follow-up time, which biases individual studies by not allowing for an accurate assessment of possible outcomes. Outcomes may thus be attributable to baseline differences and although associated with the interventions, may not necessarily be causally linked to the interventions.

Secondly, due to high heterogeneity, we were not able to complete a network meta-analysis and instead performed a narrative synthesis of results. Limitations to narrative synthesis include the potential biasing of results by overemphasizing the outcomes of particular studies, and the inability to objectively compare the different options available.

Thirdly, results primarily focused on findings comparing laparoscopic tubal ligation and/or hysteroscopic tubal occlusion, with 10 out of 24 studies assessing bilateral salpingectomy and no articles assessing the LNG-IUC. Therefore, results of studies focusing on bilateral salpingectomy may be overemphasized as our outcomes are based on less available evidence.

Full text review was primarily done by one author, with a select subsection checked for accuracy. Therefore, it is possible that errors in data collection were made. Finally, as our study did not include grey literature, we must consider publication bias which overestimates significant results within studies.

## Conclusions

High quality studies that compare traditional forms of permanent contraception, such as the laparoscopic tubal ligation, with newer alternative methods, are urgently needed to provide evidence for informed decision-making for all options available to people seeking permanent female contraception.

## Supplementary Information

**Additional file 1:** PRISMA-2009-Checklist

**Additional file 2:** Risk of Bias by study design

## Data Availability

The datasets we used and/or analysed during our review are available from the corresponding author upon reasonable request. Copies of the search strategies can be found at http://med-fom-cart-grac.sites.olt.ubc.ca/files/2016/05/Search-Strategies-Librarian-edit.docx.

## Abbreviations

MeSH: Medical subject heading
LNG-IUC: Levonorgestrel-releasing intrauterine contraceptive
NOS: Newcastle Ottawa Scale

## References

[1] Patil E, Jensen J (2015). Update on permanent contraception options for women. Curr Opin Obstet Gynecol.

[2] Alton K, Jensen J (2018). Update on permanent contraception for women. Curr Obst Gynecol Rep.

[3] Joshi R, Khadilkar S, Patel M (2015). Global trends in use of long-acting reversible and permanent methods of contraception: seeking a balance. Int J Gynaecol Obstet.

[4] Black A, Yang Q, Wu Wen S, Lalonde AB, Guilbert E, Fisher W (2009). Contraceptive use among canadian women of reproductive age: results of a National Survey. J Obstet Gynaecol Can.

[5] Daniels K, Daugherty J, Jones J, Mosher W (2015). Curent contraceptive use and variation by selected characteristics among women aged 15–44: United States, 2011–2013. Natl Health Stat Report.

[6] MedEffect Canada. Summary Safety Review - ESSURE Permanent BIrth Control System - Assessing the Risk of Complications and the Potential Need for Device Removal. In: Health Canada, ed., 2016.

[7] FDA. FDA News Release: FDA takes additional action to better understand safety of Essure, inform patients of potential risks. 2016.

[8] Bayer. Essure FAQ. Whippany, NJ: Bayer, 2018 (vol 2019).

[9] Nichols M, Carter JF, Fylstra DL, Childers M (2006). A comparative study of hysteroscopic sterilization performed in-office versus a hospital operating room. J Minim Invasive Gynecol.

[10] Kerin J, Carignan C, Cher D (2001). The safety and effectiveness of a new hysteroscopic method for permanent birth control: results of the first EssureTM pbc clinical study. Aust N Z J Obstet Gynaecol.

[11] Chapelle CF, Veersema S, Brölmann HA, Jansen FW (2015). Effectiveness and feasibility of hysteroscopic sterilization techniques: a systematic review and meta-analysis. Fertil Steril.

[12] Royal Australian and New Zealand College of Obstetricians and Gynaecologists. Managing the Adnexae at the time of Hysterectomy for Benign Gynaecological Disease, 2014.

[13] American College of Obstetricians and Gynecologists. ACOG Committee Opinion No. 774: Opportunistic salpingectomy as a strategy for epithelial ovarian cancer prevention. Obstet Gynecol 2019;133:e279-e84. 10.1097/AOG.0000000000003164 30913199

[14] Salvador S, Scott S, Francis J, Agrawal A, Giede C (2017). No. 344-Opportunistic salpingectomy and other methods of risk reduction for ovarian/fallopian tube/peritoneal cancer in the general population. J Obst Gynaecol Can.

[15] Salvador A, Gilks B, Kobel M, Huntsman D, Rosen B, Miller D (2009). The fallopian tube: primary site of most pelvic high-grade serous carcinomas. Int J Gynecol Cancer.

[16] Hanley GE, McAlpine JN, Kwon JS, Mitchell G (2015). Opportunistic salpingectomy for ovarian cancer prevention. Gynecol Oncol Res Pract.

[17] Kim AJ, Barberio A, Berens P (2019). The trend, feasibility, and safety of salpingectomy as a form of permanent sterilization. J Minim Invas Gynecol.

[18] Powell CB, Alabaster A, Simmons S (2017). Salpingectomy for sterilization: change in practice in a large integrated health care system, 2011–2016. Obstet Gynecol.

[19] Canadian Cancer Statistics Advisory Committee (2018). Canadian Cancer Statistics 2018.

[20] Dilley SE, Havrilesky LJ, Bakkum-Gamez J (2017). Cost-effectiveness of opportunistic salpingectomy for ovarian cancer prevention. Gynecol Oncol.

[21] Kwon JS, McAlpine JN, Hanley GE (2015). Costs and benefits of opportunistic salpingectomy as an ovarian cancer prevention strategy. Obstet Gynecol.

[22] Ti A, Roe A, Whitehouse K, Smith R, Gaffield M, Curtis K (2020). Effectiveness and safety of extending intrauterine device duration: a systematic review. Am J Obstet Gynecol.

[23] Kailasam C, Cahill D (2008). Review of the safety, efficacy and patient accepability of hte levonorgestrel-releasing intrauterine system. Pat Prefer Adherence.

[24] Van Houdenhoven K, van Kaam KJAF, van Grootheest AC, Salemans THB, Dunselman GAJ (2006). Uterine perforation in women using a levonorgestrel-releasing intrauterine system. Contraception.

[25] McKay R, Schunmann C (2017). Male and female sterilisation. Obstet Gynaecol Reprod Med.

[26] Gormley R, Vickers B, Norman W (2019). Comparing options for women seeking permanent contraception in high-resource countries: a protocol for a systematic review. Syst Rev.

[27] Moher D, Liberati A, Tetzlaff J, Altman D (2009). Preferred reporting items for systematic reviews and meta-analyses: the PRISMA statement. PLoS Med.

[28] Liberati A, Altman D, Tetzlaff J (2009). The PRISMA statement for reporting systematic reviews and meta-analyses of studies that evaluate health care interventions: explanation and elaboration. PLoS Med.

[29] World Bank Group. World Bank Country and Lending Groups. 2019.

[30] Wells G, Shea B, O’Connell D, et al. The Newcastle-Ottawa Scale (NOS) for assessing the quality of nonrandomised studies in meta-analyses., N.d.

[31] Guyatt GH, Oxman AD, Vist G (2011). GRADE guidelines: 4. Rating the quality of evidence–study limitations (risk of bias). J Clin Epidemiol.

[32] Ryan R. Cochrane Consumers and Comunication Review Group: data synthesis and analysis. In: Group. CCaCR, ed., 2013.

[33] Popay J, Roberts H, Sowden A, et al. Guidance on the conduct of narrative synthesis in systematic reviews. A product from the ESRC methods programme Version 2006;1:b92.

[34] Antoun L, Smith P, Gupta JK, Clark TJ (2017). The feasibility, safety, and effectiveness of hysteroscopic sterilization compared with laparoscopic sterilization. Am J Obstet Gynecol.

[35] Bouillon K, Bertrand M, Bader G, Lucot J, Dray-Spira R, Zureik M (2018). Association of hysteroscopic vs laparoscopic sterilization with procedural, gynecological, and medical outcomes. JAMA.

[36] Fernandez H, Legendre G, Blein C, Lamarsalle L, Panel P (2014). Tubal sterilization: pregnancy rates after hysteroscopic versus laparoscopic sterilization in France, 2006–2010. Eur J Obst Gynecol Reprod Biol.

[37] Jokinen E, Heino A, Karipohja T, Gissler M, Hurskainen R (2017). Safety and effectiveness of female tubal sterilisation by hysteroscopy, laparoscopy, or laparotomy: a register based study. BJOG.

[38] Mao J, Pfeifer S, Schlegel P, Sedrakyan A (2015). Safety and efficacy of hysteroscopic sterilization compared with laparoscopic sterilization: an observational cohort study. BMJ.

[39] Perkins RB, Morgan JR, Awosogba TP, Ramanadhan S, Paasche-Orlow MK (2016). Gynecologic outcomes after hysteroscopic and laparoscopic sterilization procedures. Obstet Gynecol.

[40] Conover MM, Howell JO, Wu JM, Kinlaw AC, Dasgupta N, Funk MJ (2015). Incidence of opioid-managed pelvic pain after hysteroscopic sterilization versus laparoscopic sterilization, US 2005–2012. Pharmacoepidemiol Drug Saf.

[41] Steward R, Carney P, Law A, Xie L, Wang Y, Yuce H (2018). Long-term outcomes after elective sterilization procedures - a comparative retrospective cohort study of Medicaid patients. Contraception.

[42] Carmona F, Cristobal P, Casamitjana R, Balasch J (2003). Effect of tubal sterilization on ovarian follicular reserve and function. Am J Obstet Gynecol.

[43] Gaitskell K, Coffey K, Green J (2016). Tubal ligation and incidence of 26 site-specific cancers in the Million Women Study. Br J Cancer.

[44] McAlpine JN, Hanley GE, Woo MMM (2014). Opportunistic salpingectomy: uptake, risks, and complications of a regional initiative for ovarian cancer prevention. Am J Obstet Gynecol.

[45] Hanley GE, Kwon JS, Finlayson S, Huntsman DG, Miller D, McAlpine JN (2018). Extending the safety evidence for opportunistic salpingectomy in prevention of ovarian cancer: a cohort study from British Columbia, Canada. Am J Obstet Gynecol.

[46] Malacova E, Kemp A, Hart R, Jama-Alol K, Preen DB (2014). Long-term risk of ectopic pregnancy varies by method of tubal sterilization: a whole-population study. Fertil Steril.

[47] Zerden ML, Castellano T, Doll KM, Stuart GS, Munoz MC, Boggess KA (2018). Risk-reducing salpingectomy versus standard tubal sterilization: lessons from offering women options for interval sterilization. South Med J.

[48] Westberg J, Scott F, Creinin MD (2017). Safety outcomes of female sterilization by salpingectomy and tubal occlusion. Contraception.

[49] Falconer H, Yin L, Gronberg H, Altman D (2015). Ovarian cancer risk after salpingectomy: a nationwide population-based study. JNCI J Natl Cancer Inst.

[50] Madsen C, Baandrup L, Dehlendorff C, Kjær SK (2015). Tubal ligation and salpingectomy and the risk of epithelial ovarian cancer and borderline ovarian tumors: a nation-wide case-control study. Acta Obstet Gynecol Scand.

[51] Lessard-Anderson CR, Handlogten KS, Molitor RJ (2014). Effect of tubal sterilization technique on risk of serous epithelial ovarian and primary peritoneal carcinoma. Gynecol Oncol.

[52] Mao J, Guiahi M, Chudnoff S, Schlegel P, Pfeifer S, Sedrakyan A (2019). Seven-Year outcomes after hysteroscopic and laparoscopic sterilizations. Obstet Gynecol.

[53] Carney PI, Yao J, Lin J, Law A (2017). Comparison of Healthcare costs among commercially insured women in the United States who Underwent Hysteroscopis sterilization vs laparoscopic bilateral tubal ligation sterilization. J Women’s Health.

[54] Levie MD, Chudnoff SG (2005). Office hysteroscopic sterilization compared with laparoscopic sterilization: a critical cost analysis. J Minim Invasive Gynecol.

[55] Hopkins MR, Creedon DJ, Wagie AE, Williams AR, Famuyide AO (2007). Retrospective cost analysis comparing Essure hysteroscopic sterilization and laparoscopic bilateral tubal coagulation. J Minim Invasive Gynecol.

[56] Grimes DA, Mishell DR (2008). Intrauterine contraception as an alternative to interval tubal sterilization. Contraception.

[57] Curtis K, Marchbanks P, Peterson H (2007). Neoplasia with use of intrauterine devices. Contraception.

[58] Coffey K, Beral V, Green J, Reeves G, Barnes I (2015). Lifestyle and reproductive risk factors associated with anal cancer in women aged over 50 years. Br J Cancer.

[59] Silva does Santas PN, Madden T, Omvig K, Peipert J (2017). Changes in body composition in women using long-acting reversible contraception. Contraception.

[60] Mills K, Marchand G, Sainz K (2021). Salpingectomy vs tubal ligation for sterilization: a systematic review and meta-analysis. Am J Obstet Gynecol.

[61] Hanley GE, Kwon JS, McAlpine JN, Huntsman DG, Finlayson SJ, Miller D (2020). Examining indicators of early menopause following opportunistic salpingectomy: a cohort study from British Columbia, Canada. Am J Obstet Gynecol.

[62] Madden T, McNicholas C, Zhao Q, Secura GM, Eisenberg DL, Peipert JF (2014). Association of age and parity with intrauterine device expulsion. Obstet Gynecol.

[63] Youm J, Lee HJ, Kim SK, Kim H, Jee BC (2014). Factors affecting the spontaneous expulsion of the levonorgestrel-releasing intrauterine system. Int J Gynecol Obstet.

[64] Backman T, Huhtala S, Blom T, Luoto R, Rauramo I, Koskenvuo M (2000). Length of use and symptoms associated with premature removal of the levonorgestrel intrauterine system: a nation-wide study of 17,360 users. BJOG.

[65] Carvalho NM, Chou V, Modesto W, Margatho D, Garcia EA, Bahamondes L (2017). User satisfaction with a levonorgestrel-releasing intrauterine system (LNG-IUS): data from an international survey. Obst Genecol Res.

[66] Romer T, Linsberger D (2009). User satisfaction with a levonorgestrel-releasing intrauterine system (LNG-IUS): data from an international survey. Eur J Contracept Reprod Health Care.

[67] Jensen J, Nelson A, Costales A (2008). Subject and clinician experience with the levonorgestrel-releasing intrauterine system. Contraception.

[68] Jareid M, Thalabard J, Aarflot M, Bovelstad H, Lund E, Braaten T (2018). Levonorgestrel-releasing intrauterine system use is associated with a decreased risk of ovarian and endometrial cancer, without increased risk of breast cancer. Results from the NOWAC Study. Gynecol Oncol.

[69] Tai RWM, Choi SKY, Coyte PC (2018). The cost-effectiveness of salpingectomies for family planning in the prevention of ovarian cancer. J Obstet Gynaecol Can.

