# Supplementary material for: Comparing options for females seeking permanent contraception in high resource countries: a systematic review
Source: Reprod Health. 2021 Jul 20;18:154. doi: 10.1186/s12978-021-01201-z (PMC8290533; doi:10.1186/s12978-021-01201-z)
Supplement: Supplementary file 2 — Additional file 2: Risk of Bias by study design [file 12978_2021_1201_MOESM2_ESM.docx]

| Cohort Study Design | | | | |
| --- | --- | --- | --- | --- |
| Study | Selection | Comparability | Outcome | NOS Score |
| Abbuhl, 1997 | *** |  | * | 4 |
| Antoun, 2017 | **** |  | *** | 7 |
| Bouillon, 2018 | **** | ** | *** | 9 |
| Carney, 2017 | **** | ** | *** | 9 |
| Conover, 2015 | **** | ** | ** | 8 |
| Duffy, 2005 | *** | ** | * | 6 |
| Falconer, 2015 | **** | ** | *** | 9 |
| Fernandez, 2014 | **** | * | ** | 7 |
| Gaitskell, 2016 | **** | ** | *** | 9 |
| Hanley, 2018 | **** | ** | ** | 8 |
| Hopkins, 2007 | **** | * | ** | 7 |
| Jokinen, 2017 | **** | * | ** | 7 |
| Kim, 2019 | **** | ** | *** | 9 |
| Malacova, 2014 | **** | * | *** | 8 |
| Mao, 2019 | **** | ** | *** | 9 |
| Mao, 2015 | **** | ** | *** | 9 |
| McAlpine, 2014 | **** | * | ** | 7 |
| Niblock, 2014 | *** |  | * | 4 |
| Perkins, 2016 | **** | * | *** | 8 |
| Powell, 2017 | **** |  | *** | 7 |
| Rulin, 1993 | ** |  | ** | 4 |
| Steward, 2017 | **** | * | *** | 8 |
| Syed, 2007 | ** |  | * | 3 |
| Theil, 2008 | ** |  | ** | 4 |
| Trussel, 1995 | ** | * | * | 4 |
| Westberg, 2017 | **** |  | *** | 7 |
| Zerden, 2018 | *** | * | *** | 7 |

**Risk of Bias of Included Articles using the Newcastle Ottawa Scale (NOS) included in the Comparing Female Permanent Contraception Options in High Resource Countries: A Systematic Review: Cohort Study Design**

**Risk of Bias of Included Articles using the Newcastle Ottawa Scale (NOS) included in the Comparing Female Permanent Contraception Options in High Resource Countries: A Systematic Review: Case-Control Design**

| Case Control Study Design | | | | |
| --- | --- | --- | --- | --- |
| Study | Selection | Comparability | Outcome / Exposure | NOS Score |
| Carmona, 2003 | *** | * | *** | 7 |
| Franchini, 2009 | ** | ** | * | 5 |
| Greisman, 1991 | ** |  | ** | 4 |
| Kjer, 1990 | ** |  | ** | 4 |
| Lessard-Anderson, 2014 | **** | ** | *** | 9 |
| Levie, 2005 | *** | * | *** | 7 |
| Madsen, 2015 | **** | ** | *** | 9 |
